# Establishment and validation of a novel nomogram incorporating clinicopathological parameters into the TNM staging system to predict prognosis for stage II colorectal cancer

**DOI:** 10.1186/s12935-020-01382-w

**Published:** 2020-07-06

**Authors:** Shaobo Mo, Zheng Zhou, Yaqi Li, Xiang Hu, Xiaoji Ma, Long Zhang, Sanjun Cai, Junjie Peng

**Affiliations:** 1 Department of Colorectal Surgery, Fudan University Shanghai Cancer Center, 270 Dong’an Road, Shanghai, 200032 China; 2 Department of Oncology, Shanghai Medical College, Fudan University, Shanghai, 200032 China; 3 Department of Cancer Institute, Fudan University Shanghai Cancer Center, Fudan University, Shanghai, 200032 China

**Keywords:** Nomogram, Colorectal cancer, Stage II, Prognosis, Clinical utility

## Abstract

**Background:**

Survival outcomes are significantly different in stage II colorectal cancer (CRC) patients with diverse clinicopathological features. The objective of this study is to establish a credible prognostic nomogram incorporating easily obtained parameters for stage II CRC patients.

**Methods:**

A total of 1708 stage II CRC patients seen at Fudan University Shanghai Cancer Center (FUSCC) from 2008 to 2013 were retrospectively analyzed in this study. Cases were randomly separated into a training set (n = 1084) and a validation set (n = 624). Univariate and multivariate Cox regression analyses were used to identify independent prognostic factors that were subsequently incorporated into a nomogram. The performance of the nomogram was evaluated by the predicted concordance index (C-index) and ROC curve to calculate the area under the curve (AUC). The clinical utility of the nomogram was evaluated using decision curve analysis (DCA).

**Results:**

In univariate and multivariate analyses, eight parameters were correlated with disease-free survival (DFS), which were subsequently selected to generate a prognostic nomogram based on DFS. For DFS predictions, the C-index values of the nomogram were 0.842 (95% confidence interval (CI) 0.710–0.980), and 0.701 (95% CI 0.610–0.770) for the training and validation sets, respectively. The AUC values of the ROC curves for the nomogram to predicted 1, 3 and 5-year survival were 0.869, 0.858, and 0.777 (training group) and 0.673, 0.714, and 0.706 (validation group), respectively. The recurrence probability calibration curve showed good consistency between actual observations and nomogram-based predictions. DCA showed better clinical application value for the nomogram than the TNM staging system.

**Conclusion:**

A novel nomogram was established and validated in a large population, and the nomogram is a simple-to-use tool for physicians to facilitate postoperative personalized prognostic evaluation and determine therapeutic strategies for stage II CRC patients.

## Background

Colorectal cancer (CRC) is the most common malignant tumor of the digestive system and was the fourth leading cause of cancer death in China 2017 [1]. The prognosis of CRC is associated with the American Joint Commission on Cancer/International Union against Cancer (AJCC/UICC) tumor-node-metastasis (TNM) staging system. According to the TNM staging system, approximately one-quarter of CRC patients are diagnosed with stage II disease, approximately 25% of whom suffer from disease relapse after surgery [2]. However, prognosis is obviously divergent in CRC patients even with the same TNM stage due to substantial disease heterogeneity, especially for stage II CRC. Previous research showed that the outcomes in AJCC/UICC stage II CRC patients varied from close to those of stage I patients in terms of relapse and survival to being worse than those of patients with node-positive tumors [3, 4]. Therefore, the TNM staging system is not always able to accurately predict the prognosis of stage II CRC patients. Accurate postoperative personalized prognostic evaluation for patients with stage II CRC is an important step for physicians to better determine therapeutic strategies.

Clinically, whether to undergo or forego adjuvant chemotherapy has been controversial for decades, which has resulted in overtreatment and undertreatment for stage II CRC patients. Traditionally, clinicopathological features related to recurrence in stage II tumors, such as T4 lesions [5], poor histological differentiation [6], perineural invasion [7] and so on, have been identified and recommended as evidence for adjuvant chemotherapy [8, 9]. However, the results were still unsatisfactory [10, 11]. Currently, microsatellite instability (MSI) and mismatch repair deficiency (dMMR) are the most important biomarkers and are widely used to help physicians choose adjuvant chemotherapy and predict patient outcomes in stage II CRC patients [12]. Unfortunately, most stage II CRCs are classified as being microsatellite stable (MSS) or having proficient MMR (pMMR), and biomarkers are lacking for these patients. Moreover, these clinicopathological features do not clearly distinguish between patients who have a high or low risk of disease recurrence. Thus, there is a dire need to add prognostic and predictive values to the current TNM staging system with the purpose of determining those patients more likely to suffer from tumor relapse.

Several studies have tried to improve postoperative risk stratification and prediction of chemotherapy benefit for stage II CRC. Zhang et al. [13] identified a six-miRNA-based classifier that is a reliable tool for predicting prognosis and disease recurrence in patients with stage II colon cancer. Gao et al. [14] identified eight cancer hallmark-based gene signatures (30 genes each) used them to determine prognosis in stage II CRC. Despite effective risk stratification in stage II CRC, application of the identified signatures exacerbated the financial burden on patients, and the signatures remain far from application in clinical practice.

Therefore, we aimed to establish a simple-to-use and personalized scoring system meeting clinicians’ needs to predict the prognosis of stage II CRC. In the current study, information on stage II CRC diagnosed at Fudan University Shanghai Cancer Center (FUSCC) was extracted to construct and validate a nomogram to predict patient prognosis, which was subsequently proven to have strong clinical application value by decision curve analysis (DCA).

## Methods

### Ethics statement

The Ethical Committee and Institutional Review Board of the Fudan University Shanghai Cancer Center reviewed and approved this study protocol. All patients signed written informed consent.

### Patients

**Fig. 1:**
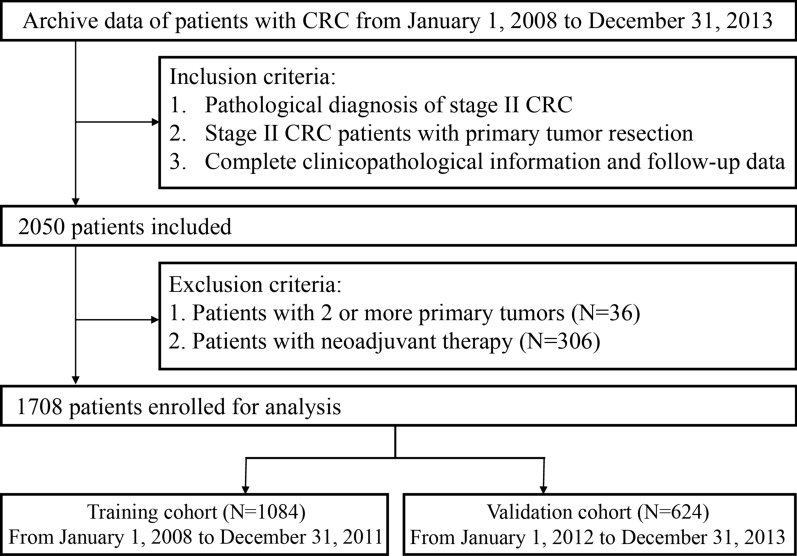
Recruitment pathway of stage II CRC patients in this study

A total of 1708 patients with stage II CRC diagnosed and undergoing radical surgery at FUSCC from January 1, 2008, to December 31, 2013, were retrospectively reviewed. We recruited patients meeting the following criteria: (1) patients with a pathological diagnosis of stage II CRC; (2) stage II CRC patients with primary tumor resection performed at our center; and (3) patients with complete clinicopathological information and follow-up data. Patients who met the following exclusion criteria were excluded: (1) patients who accepted neoadjuvant therapy and (2) patients who had multiple primary tumors. All eligible patients were regrouped according to the 8^th^ AJCC/UICC TNM staging system. The detailed workflow for patient selection is shown in Fig. 1.

Fifteen variables were extracted from FUSCC in this study, including pretreatment carcinoembryonic antigen (Pre-CEA) level, age, sex, adjuvant chemotherapy, lymphovascular invasion, perineural invasion, circumferential resection margin (CRM) status, tumor size, number of lymph nodes harvested (LNH), histological type, family history, tumor site, mismatch repair (MMR) status, histological differentiation, and T stage. Patients were separated into a training group (n = 1084, from January 1, 2008, to December 31, 2011) and a validation group (n = 624, from January 1, 2012, to December 31, 2013).

### Construction and validation of the nomogram

Univariate and multivariate analyses were conducted via the Cox regression method to identify independent risk factors in the training cohort. Based on multivariate Cox regression analyses, a simple-to-use nomogram incorporating seven clinicopathological parameters into the TNM staging system was formulated. The total points for each patient in the validation group were calculated using the established nomogram, after which a Cox regression analysis of the whole cohort was performed using the total points as a parameter. The 1-, 3-, and 5-year calibration plots graphically show the relationship between the predicted and observed risk for each outcome to assess the predictive ability of the nomogram.

### Concordance index (C-index), receiver operating characteristic (ROC) curve and DCA

The C-index and ROC curve methods were used to appraise the discriminating ability of the nomogram. The C-index was defined as the ratio of all patient pairs whose predictions were consistent with the results. The 1-, 3-, and 5-year ROC curves were used to evaluate the nomogram’s predictive ability for different time periods. DCA was recently proposed as a novel method for evaluating predictive models and visualizing the clinical consequences of a treatment strategy [15], and it was carried out to determine the potential benefit of the predictive nomogram in this study.

### Risk stratification based on the novel nomogram

To reveal the independent discrimination ability of the simple-to-use prognostic nomogram, we regrouped all patients into high-, moderate-, and low-risk groups according to the total risk scores in the study cohort. Survival curves for different risk groups were generated using the Kaplan–Meier method and were compared using the log-rank test.

### Statistical analyses

The R software was used for the randomization of patients. The Chi-square test was used to compare the differences between the training and validation groups for the categorical variables. The R statistical packages “rms”, “survival”, “Hmisc”, “MASS”, “survivalROC” and “rmda” were used to calculate the C-index, plot the calibration and ROC curves, build a nomogram, and draw the DCA curves and Kaplan–Meier curves. All statistical analyses were performed using R (version 2.15.0, http://www.r-project.org). All statistical tests were 2-sided, and p values < 0.05 were considered statistically significant.

## Results

### Demographic and clinical characteristics

A total of 1708 patients with stage II CRC were retrospectively collected from the institutional database. The clinicopathological characteristics and demographics of the entire (N = 1708), training (N = 1084), and validation (N = 624) cohorts are listed in Table 1.

**Table 1:** Demographics and clinical characteristics of eligible patients with stage II CRC

Characteristics	All patients	Training group	Validation group	p value
	N = 1708	N = 1084	N = 624	
Gender, n (%)				0.157
Female	645 (37.8)	423 (39.0)	222 (35.6)	
Male	1063 (62.2)	661 (61.0)	402 (64.4)	
Age, n (%)				0.883
< 60	806 (47.2)	513 (47.3)	293 (47.0)	
≥ 60	902 (52.8)	571 (52.7)	331 (53.0)	
Pre-CEA, n (%)				0.226
Negative	1121 (65.6)	700 (64.6)	421 (67.5)	
Positive	587 (34.4)	384 (35.4)	203 (32.5)	
Family history, n (%)				0.480
No	1171 (68.6)	732 (67.5)	439 (70.4)	
Yes	429 (25.1)	281 (26.0)	148 (23.7)	
Unknown	108 (6.3)	71 (6.5)	37 (5.9)	
Adjuvant CT, n (%)				0.318
No	476 (27.9)	311 (28.7)	165 (26.4)	
Yes	1232 (72.1)	773 (71.3)	459 (73.6)	
Tumor site, n (%)				0.414
Left	1206 (70.6)	758 (69.9)	448 (71.8)	
Right	502 (29.4)	326 (30.1)	176 (28.2)	
T stage, n (%)				0.219
T3	1124 (65.8)	697 (64.3)	427 (68.4)	
T4a	550 (32.2)	365 (33.7)	185 (29.7)	
T4b	34 (2.0)	22 (2.0)	12 (1.9)	
Histological type, n (%)				0.585
Adenocarcinoma	1386 (81.1)	877 (80.9)	509 (81.6)	
Mucinous adenocarcinoma	316 (18.5)	202 (18.6)	114 (18.3)	
Signet-ring cell carcinoma	6 (0.4)	5 (0.5)	1 (0.1)	
Histological differentiation, n (%)				0.840
Well	143 (8.4)	94 (8.7)	49 (7.8)	
Moderate	1349 (79.0)	853 (78.7)	496 (79.5)	
Poor	216 (12.6)	137 (12.6)	79 (12.7)	
Tumor size, n (%)				0.421
< 4	614 (35.9)	382 (35.2)	232 (37.2)	
≥ 4	1094 (64.1)	702 (64.8)	392 (62.8)	
LNH, n (%)				0.495
< 12	296 (17.3)	193 (17.8)	103 (16.5)	
≥ 12	1412 (82.7)	891 (82.2)	521 (83.5)	
Lymphovascular invasion, n (%)				0.943
Negative	1534 (89.8)	974 (89.9)	560 (89.7)	
Positive	174 (10.2)	110 (10.1)	64 (10.3)	
Perineural invasion, n (%)				0.351
Negative	1463 (85.7)	922 (85.1)	541 (86.7)	
Positive	245 (14.3)	162 (14.9)	83 (13.3)	
CRM, n (%)				0.429
Negative	1688 (98.8)	1073 (99.0)	615 (98.6)	
Positive	20 (1.2)	11 (1.0)	9 (1.4)	
MMR status, n (%)				0.602
dMMR	456 (26.7)	294 (27.1)	162 (26.0)	
pMMR	1252 (73.3)	790 (72.9)	462 (74.0)	
Tumor stage, n (%)				0.219
Stage IIA	1124 (65.8)	697 (64.3)	427 (68.4)	
Stage IIB	550 (32.2)	365 (33.7)	185 (29.7)	
Stage IIC	34 (2.0)	22 (2.0)	12 (1.9)	

*CRC* colorectal cancer, *Pre*-*CEA* pretreatment carcinoembryonic antigen, *CT* chemotherapy, *LNH* number of lymph nodes harvested, *CRM* circumferential resection margin, *MMR* mismatch repair, *dMMR* deficient mismatch repair, pMMR, proficient mismatch repair

In the entire group, 62.2% of patients were male, and 47.2% of patients were < 60 years at diagnosis. Most patients had an adenocarcinoma histological type, moderately differentiated tumors, and LNH ≥ 12. T3, T4a, and T4b tumors accounted for 65.8%, 32.2%, and 2.0% of all cases, respectively. Across the entire study population, a total of 72.1% of patients underwent adjuvant chemotherapy with 5-Fu-based monotherapy or combined therapy. The 5-year disease-free survival (DFS) rate was 75.9% for all patients, with a median follow-up time of 68.1 months. There was no significant difference between the training and validation cohorts in demographic and clinical characteristics.

### Independent prognostic factors in stage II CRC patients

**Fig. 2:**
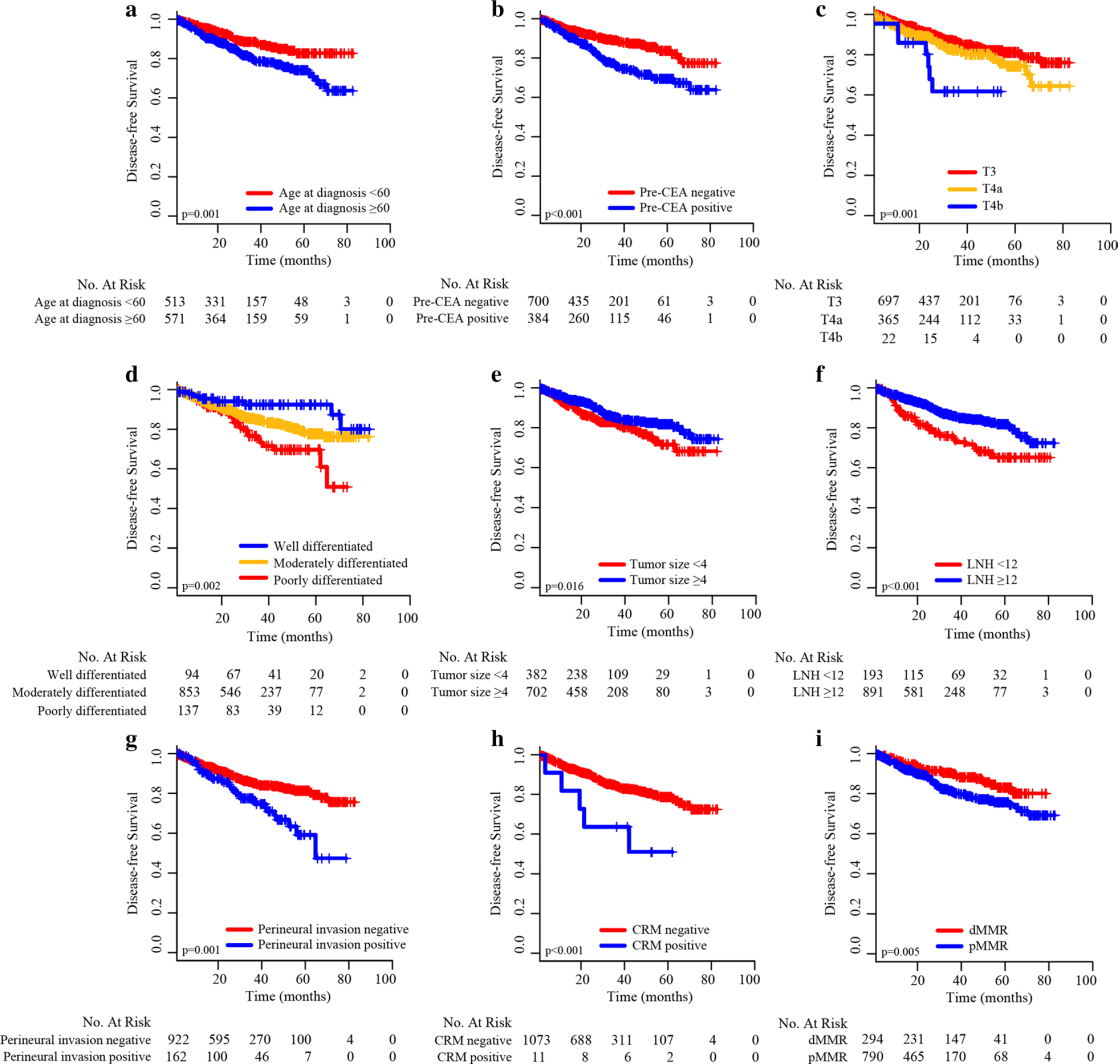
Kaplan–Meier DFS curves stratified by patients’ characteristics in the training cohort: **a** Age at diagnosis; **b** Pre-CEA; **c** T stage; **d** histological differentiation; **e** TUMOR size; **f** LNH; **g** perineural invasion; **h** CRM status; **i** MMR status

According to the results of the univariate Cox regression analysis, nine variables, age at diagnosis, pre-CEA level, T stage, histological differentiation, tumor size, LNH, perineural invasion, CRM status, and MMR status, were associated with DFS (Table 2). The Kaplan–Meier curves showed that the nine factors were related to DFS (p < 0.05, Fig. 2). In the multivariate Cox regression analysis, eight parameters, pre-CEA, age, T stage, histological differentiation, LNH, perineural invasion, CRM status, and MMR status, were defined as independent prognostic factors of stage II CRC (Table 2).

**Table 2:** Univariable and multivariable Cox regression analyses of prognostic factors in stage II CRC patients

Variables	Univariable analyses		Multivariable analyses	
	HR (95% CI)	p value	HR (95% CI)	p value
Gender		0.291		
Female	Reference			
Male	1.278 (0.811–2.014)			
Age		0.001		0.007
< 60	Reference		Reference	
≥ 60	2.254 (1.402–3.623)		1.615 (1.138–2.292)	
Pre-CEA		0.008		0.002
Negative	Reference		Reference	
Positive	1.795 (1.165–2.764)		1.678 (1.210–2.325)	
Family history		0.739		
No	Reference			
Yes	1.278 (0.463–3.523)	0.500		
Unknown	1.076 (0.369–3.140)	0.636		
Adjuvant CT		0.793		
No	Reference			
Yes	1.064 (0.669–1.694)			
Tumor site		0.367		
Left	Reference			
Right	0.901 (0.719–1.130)			
T stage		0.002		0.003
T3	Reference		Reference	
T4a	1.358 (0.975–1.891)	0.070	1.419 (1.005–2.002)	0.047
T4b	3.350 (1.619–6.932)	0.001	3.221 (1.532–6.776)	0.002
Histological type		0.244		
Adenocarcinoma	Reference			
Mucinous adenocarcinoma	1.019 (0.675–1.537)	0.929		
Signet-ring cell carcinoma	3.320 (0.819–13.454)	0.093		
Histological differentiation		0.002		0.009
Well	Reference		Reference	
Moderate	3.428 (1.570–7.483)	0.002	2.814 (1.274–6.218)	0.009
Poor	1.965 (0.956–4.037)	0.066	1.619 (0.785–3.342)	0.192
Tumor size		0.016		0.061
< 4	Reference		Reference	
≥ 4	0.675 (0.490–0.931)		0.721 (0.512–1.015)	
LNH		< 0.001		0.012
< 12	Reference		Reference	
≥ 12	0.499 (0.353–0.705)		0.624 (0.432–0.902)	
Lymphovascular invasion		0.053		
Negative	Reference			
Positive	1.539 (0.995–2.380)			
Perineural invasion		0.001		0.029
Negative	Reference		Reference	
Positive	1.868 (1.284–2.717)		1.557 (1.048–2.315)	
CRM		0.001		0.027
Negative	Reference		Reference	
Positive	3.968 (1.752–8.990)		2.734 (1.119–6.677)	
MMR status		0.006		0.005
dMMR	Reference		Reference	
pMMR	1.705 (1.165–2.494)		1.739 (1.184–2.554)	
Tumor stage		0.002		0.003
Stage IIA	Reference		Reference	
Stage IIB	1.358 (0.975–1.891)	0.070	1.419 (1.005–2.002)	0.047
Stage IIC	3.350 (1.619–6.932)	0.001	3.221 (1.532–6.776)	0.002

*CRC* colorectal cancer, *HR* hazard ratio, *CI* confidence interval, *Pre*-*CEA* pretreatment carcinoembryonic antigen, *CT* chemotherapy, *LNH* number of lymph nodes harvested, *CRM* circumferential resection margin, *MMR* mismatch repair, *dMMR* deficient mismatch repair, *pMMR* proficient mismatch repair

### Construction and validation of the prognostic prediction nomogram

**Fig. 3:**
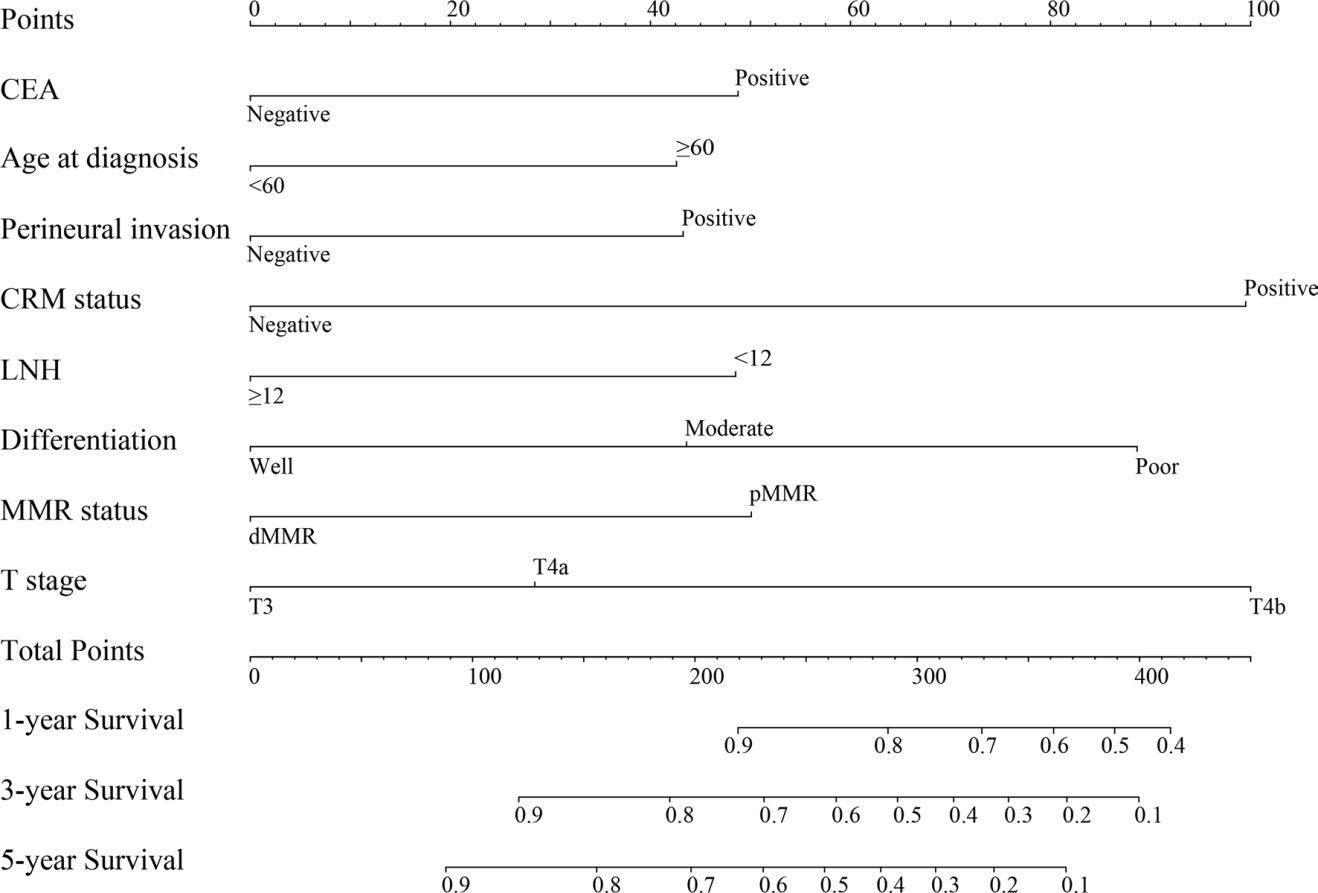
Nomograms convey the results of prognostic models using eight clinicopathological characteristics to predict DFS of patients with stage II CRC

Based on the multivariate Cox regression analysis results, pre-CEA, age, T stage, histological differentiation, LNH, perineural invasion, CRM status, and MMR status were defined as independent prognostic factors, and these were integrated to develop the nomogram (Fig. 3). According to the nomogram, T stage had the greatest influence on the prognosis of stage II CRC, followed by CRM status. Clinicians could determine the total score according to the individual scores of those eight parameters and obtain a particular probability of 1-, 3-, and 5-year DFS. Detailed scores of sub classification of each variable are listed in Additional file 1: Table S1.

**Fig. 4:**
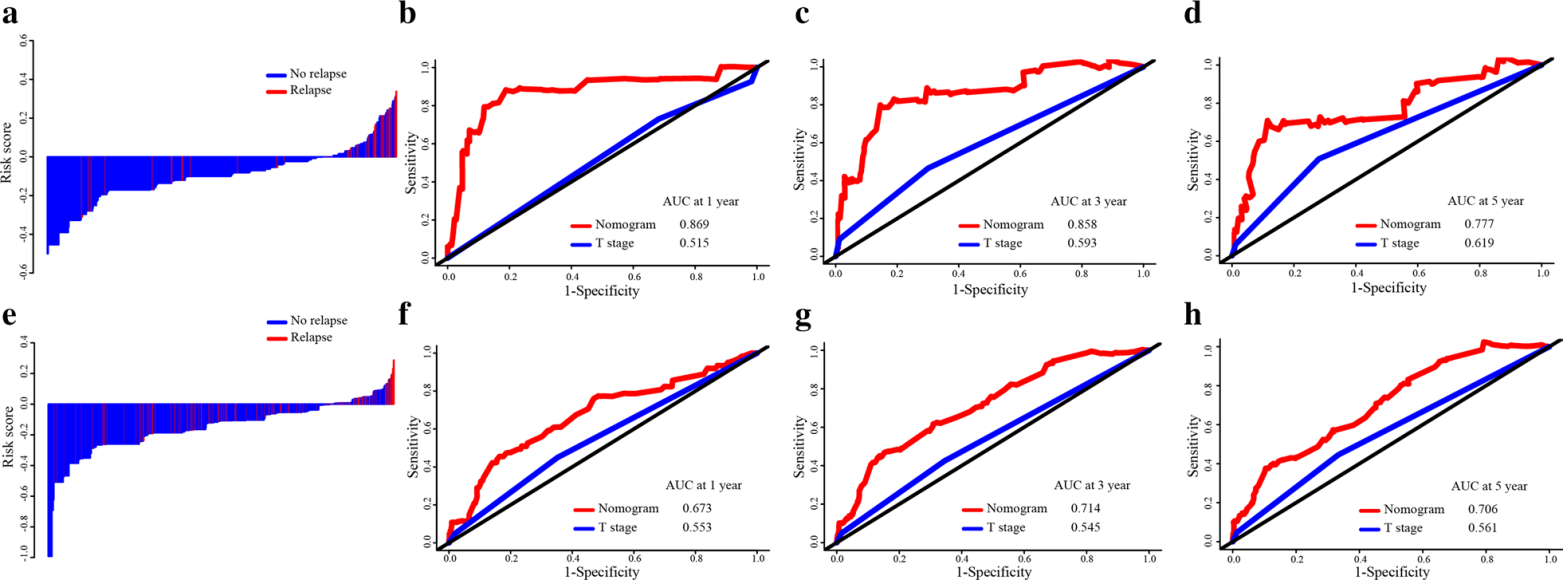
**a** Distribution of risk score and relapse status in the training cohort. **b** AUC values of ROC predicted 1-year DFS rates of Nomogram and T stage in the training cohort. **c** AUC values of ROC predicted 3-year DFS rates of Nomogram and T stage in the training cohort. **d** AUC values of ROC predicted 5-year DFS rates of Nomogram and T stage in the training cohort. **e** Distribution of risk score and relapse status in the validation cohort. **f** AUC values of ROC predicted 1-year DFS rates of Nomogram and T stage in the validation cohort. **g** AUC values of ROC predicted 3-year DFS rates of Nomogram and T stage in the validation cohort. **h** AUC values of ROC predicted 5-year DFS rates of Nomogram and T stage in the validation cohort

**Fig. 5:**
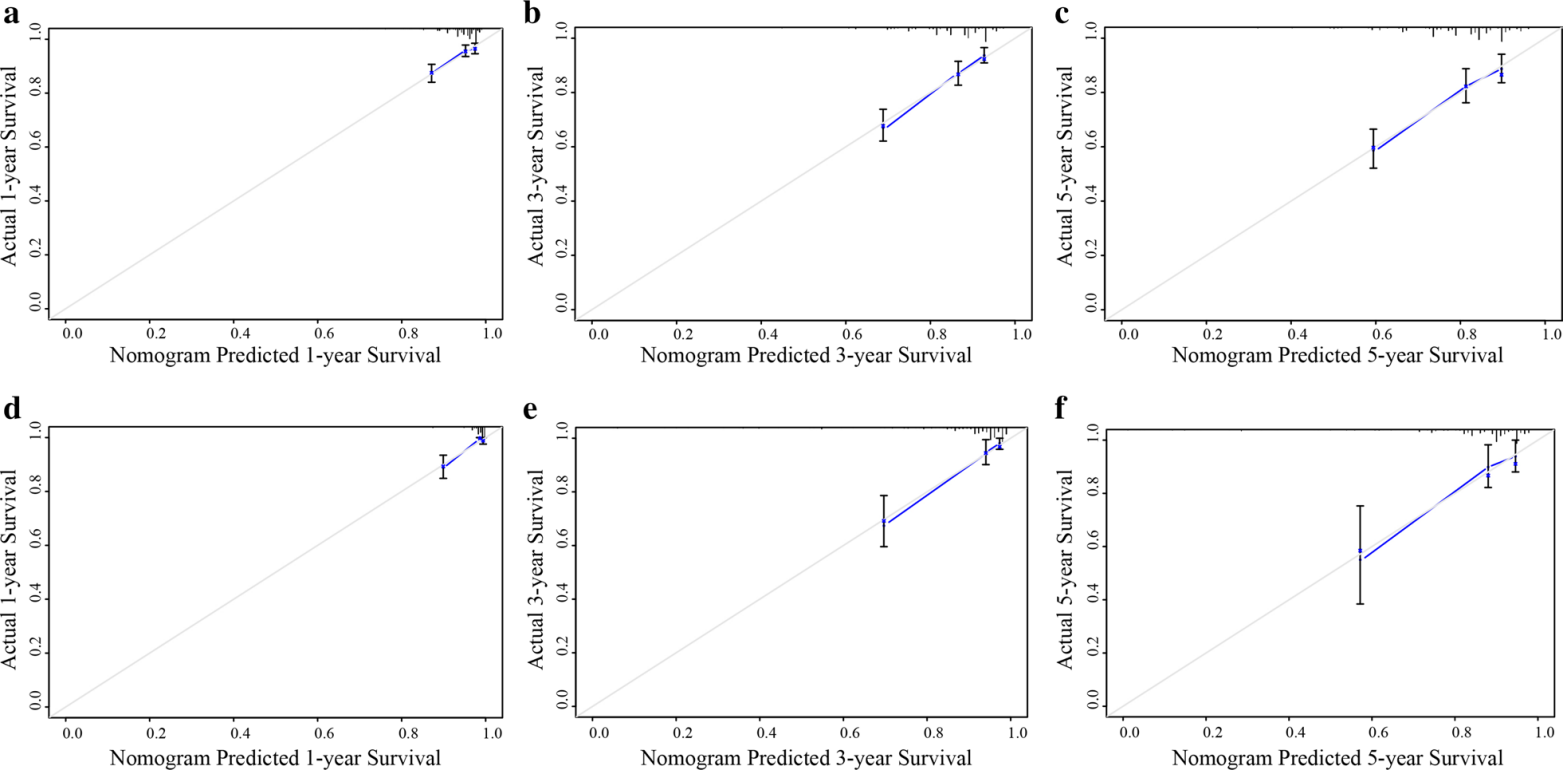
**a** The calibration curve for predicting patients’ DFS at 1-year in the training cohort. **b** The calibration curve for predicting patients’ DFS at 3-year in the training cohort. **c** The calibration curve for predicting patients’ DFS at 5-year in the training cohort. **d** The calibration curve for predicting patients’ DFS at 1-year in the validation cohort. **e** The calibration curve for predicting patients’ DFS at 3-year in the validation cohort. **f** The calibration curve for predicting patients’ DFS at 5-year in the validation cohort

The distributions of risk scores and relapse status are shown in Fig. 4a, e, and the results showed that patients with low risk scores generally had better DFS than those with high risk scores. The C-index values and ROC curves were used to evaluate the discrimination power of the nomogram. The C-indexes for the prediction of DFS in the training and validation groups were 0.842 (95% CI 0.710–0.980) and 0.701 (95% CI 0.610–0.770), respectively. To confirm that the nomogram prediction model had higher efficacy in predicting the prognosis of stage II CRC patients than T stage, time-dependent ROC analyses at 1-, 3-, and 5-year were conducted. The 1-, 3-, and 5-year AUCs of the nomogram in the training and validation groups were 0. 869, 0.858, and 0.777 and 0.673, 0.714, and 0.706, respectively, compared with AUCs of 0.515, 0.593, and 0.619 and 0.553, 0.545, and 0.561, respectively, for T stage (Fig. 4b–d, f–h), which showed that the simple-to-use nomogram incorporating clinicopathological parameters into the TNM staging system was expected to be more accurate than TNM stage. In addition, calibration curves for the nomogram showed no deviations from the reference line, which meant a high degree of credibility (Fig. 5).

### Independent prognostic performance of the nomogram in predicting prognosis in stage II CRC

**Fig. 6:**
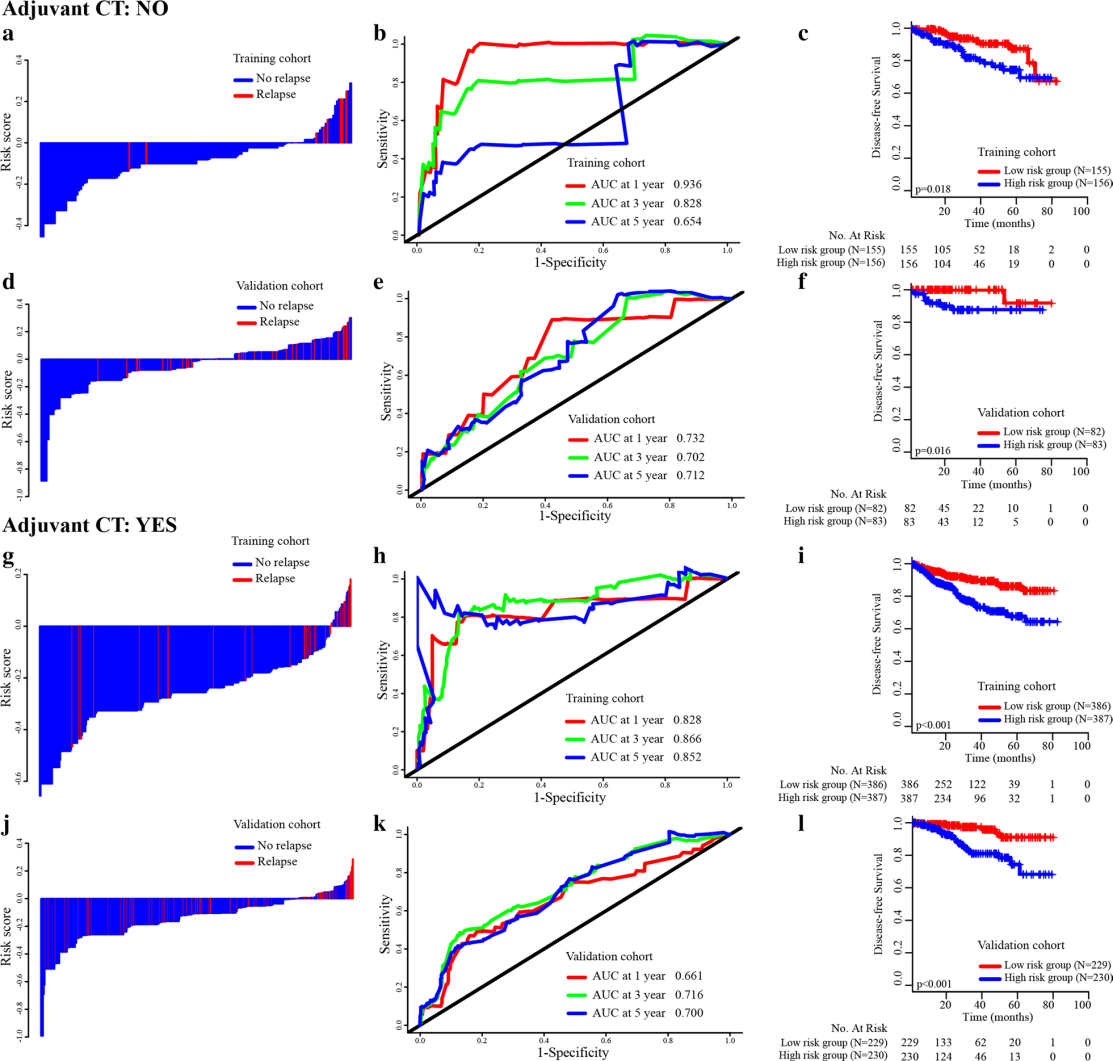
Distribution of risk score, time dependent ROC curves at 1, 3 and 5 years and Kaplan–Meier survival analysis between low and high risk patients with or without adjuvant chemotherapy in the training set (**a**–**c** and **g**–**i**) and validation set (**d**–**f** and **j**–**l**)

Whether to use adjuvant chemotherapy for stage II CRC has been controversial for decades. Subgroup analyses based on adjuvant chemotherapy suggested that the high-risk patients in each subgroup were inclined to have significantly unfavorable DFS (Fig. 6). The distributions of risk score and relapse status among each subgroup are shown in Fig. 6a, d, g, j. Time-dependent ROC analyses at 1, 3 and 5 years were conducted to assess the prognostic accuracy of the nomogram in different subgroups based on adjuvant chemotherapy (Fig. 6b, e, h, k). Patients from the training and validation cohorts were separated into a low-risk group and a high-risk group. Patients in the high-risk group tended to have poorer outcomes than those in the low-risk group, regardless of the status of adjuvant chemotherapy (Fig. 6c, f, i, l). In addition, subgroup analyses were performed based on different risk factors (LNH, perineural invasion, T stage and MMR status) and demonstrated excellent independence and prognostic value of the nomogram (Additional file 2: Figure S1).

### Clinical value of the nomogram

**Fig. 7:**
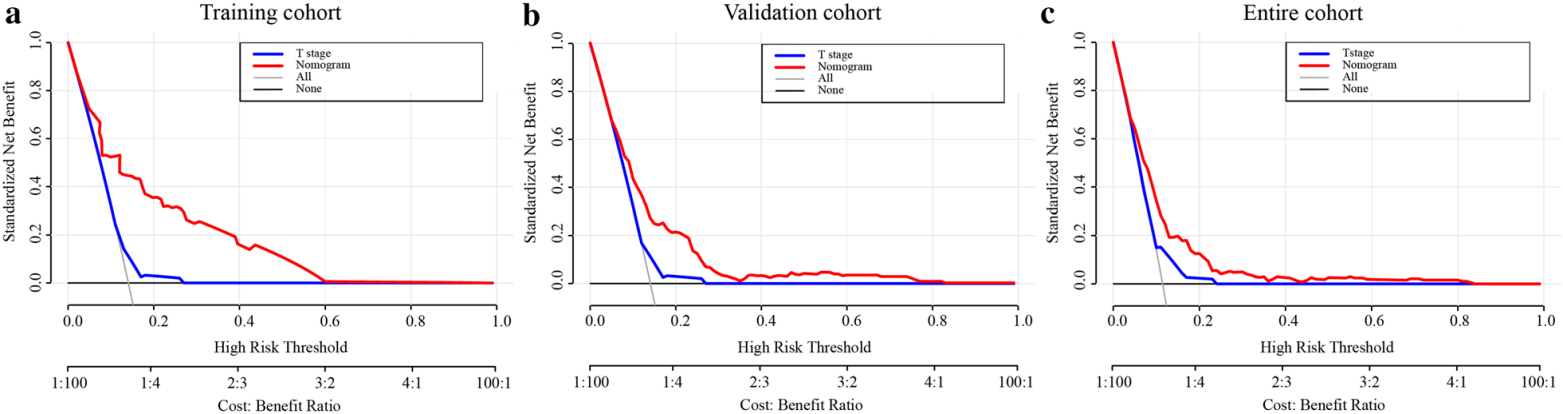
**a** Decision curve analysis of the nomogram and T stage for the survival prediction of stage II CRC patients in the training cohort. **b** Decision curve analysis of the nomogram and T stage for the survival prediction of stage II CRC patients in the validation cohort. **c** Decision curve analysis of the nomogram and T stage for the survival prediction of stage II CRC patients in the entire cohort

DCA is a novel method for evaluating alternative prognostic strategies, which has advantages over AUC. DCA curves for the novel nomogram and T stage in the training, validation and entire groups are presented in Fig. 7. Compared with that of T stage, DCA of the nomogram had higher net benefits, which indicated that the nomogram had better clinical utility than T stage.

### Prognostic nomogram for risk stratification

**Fig. 8:**
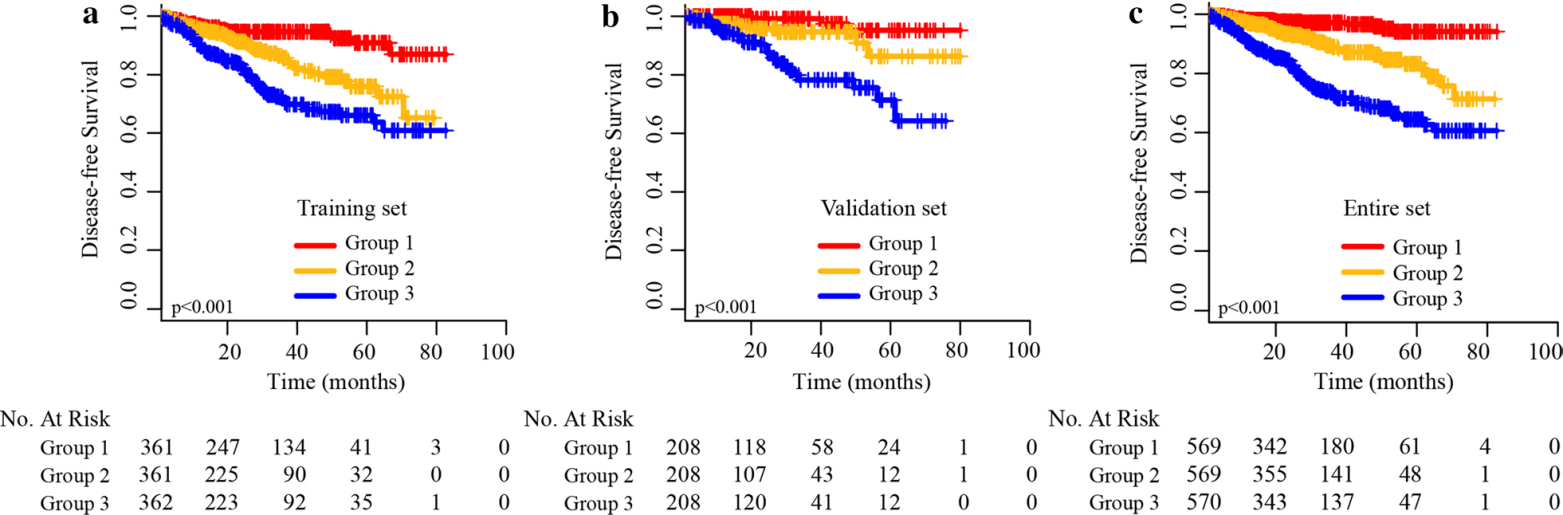
**a** Disease-free survival in the subgroups according to a tertiles of the total score from the training cohort. **b** Disease-free survival in the subgroups according to a tertiles of the total score from the validation cohort. **c** Disease-free survival in the subgroups according to a tertiles of the total score from the entire cohort

We determined the cut-off values by regrouping all patients in the training, validation and entire cohorts into three subgroups based on the total scores, and each group represented a distinct prognosis. The Kaplan–Meier survival curves were subsequently delineated and are shown in Fig. 8. In the training, validation and entire cohorts, group 1 (low-risk group) had the highest 5-year DFS at 90.9%, 95.2% and 94.1%, respectively, followed by group 2 (Moderate-risk group) at 75.9%, 86.3% and 83.3%, respectively; Group 3 (High-risk group) showed the lowest 5-year DFS for the training, validation, and entire cohorts: 66.1%, 71.4% and 67.3%, respectively. Significant statistical differences in survival outcomes were observed between the three groups.

## Discussion

In this study, a nomogram incorporating clinicopathological parameters into the TNM staging system was established to evaluate the definite 1-, 3-, and 5-year DFS probabilities of stage II CRC patients. The discriminatory ability of the nomogram was calibrated and verified in both training and validation groups. Furthermore, the nomogram was fully based on clinicopathological parameters of routine clinical operation, which made it simple to use. From the perspective of clinical usage, the nomogram had a wide scope of threshold probabilities. In addition, by comparing the AUC of the nomogram with that of T stage for predicting 1-, 3-, and 5-year DFS in the training and validation cohorts, the novel nomogram had a stronger ability to accurately reflect the exact survival probability in stage II CRC. Moreover, the nomogram was capable of dividing patients with stage II CRC into low-, moderate-, and high-risk groups, which indicated that the nomogram could be applied as a conventional tool in predicting the prognosis of stage II CRC.

In the present study, the prognosis of patients with stage II CRC was better in younger patients. Previous research has revealed that age is an independent prognostic factor of stage II CRC patients, with younger age being related to a better outcome [16]. In addition, CEA level was a potential prognostic factor in this study [17]. CEA is a well-established biomarker for CRC recommended by both the American Society of Clinical Oncology (ASCO) [18] and the European Group on Tumor Markers (EGTM) [19, 20]. Preoperative CEA levels were used to predict prognosis, and routine CEA monitoring during the postresection follow-up period was used to monitor local recurrence and distant metastases after surgery in CRC patents. As this nomogram showed, stage II CRC patients with high CEA levels tend to have significantly worse DFS rates than those with low CEA levels.

Whether adjuvant chemotherapy should be used for stage II CRC is still controversial. According to NCCN guidelines, patients with stage II CRCs and risk factors are recommended to receive adjuvant chemotherapy [8]. In this study, we performed subgroup analyses in stage II patients treated with or without adjuvant chemotherapy, and the results demonstrated the excellent independence and prognostic value of the nomogram. In the current study, histological differentiation, perineural invasion, CRM status, LNH less than 12, and T4 stage were identified as independent risk factors for stage II CRC. Histological differentiation was identified as an important feature for evaluating the benefit of adjuvant chemotherapy [21]. Our study showed that poor histological differentiation was associated with a worse prognosis. Additionally, in most studies reporting perineural invasion, similar to this research, positive perineural invasion in stage II CRC patients has been shown to be associated with poor outcomes, and such patients might thus have a greater benefit from adjuvant chemotherapy than patients without perineural invasion. Moreover, perineural invasion was defined as a major prognostic and predictive factor for response to adjuvant chemotherapy in stage II CRC [22]. CRM status is considered a significant factor for surgery quality. In our study, only 1.2% of stage II CRCs were defined as CRM positive. Despite the low percentage of patients with a positive CRM status, a positive CRM status was strongly associated with an inferior prognosis. According to the results from a population-based study, Huang et al. found that a positive CRM status decreased overall survival and cause-specific survival. The farther the CRM was from the tumor lesion, the better the long-term survival [23]. Furthermore, the appropriate staging of CRC requires at least 12 lymph nodes to be sampled, as recommended by NCCN guidelines. Relevant research indicated that stage II CRC patients with LNH less than 12 tended to have shorter DFS than those with LNH more than 12, which proved the results of this nomogram [24].

Patients with stage II CRC have differences in T stage, including patients with T3, T4a, and T4b disease. Stage T3 indicates that the cancer has grown into the outermost layers of the colon or rectum but has not gone through them. Stage T4a means that the cancer has grown through the wall of the colon or rectum but has not grown into other nearby tissues or organs while T4b means that the tumor is attached to or has grown into other nearby tissues or organs [25]. It is widely accepted that a higher T stage leads to a worse prognosis, which was duplicated in our nomogram. It is worth noting that the T stage was shown to have a strong influence on the nomogram that we established and added to its ability to predict patient risk, and the ROC analysis and DCA indicate that our nomogram has better clinical value than the TNM staging system.

Clinically, MSI or dMMR status are the most important biomarkers in stage II CRC and are widely used to help clinicians choose adjuvant chemotherapy and predict patient outcomes. Stage II CRC patients with dMMR status were more likely to have low recurrence rates and a better prognosis than those with pMMR status [25]. Clinical trials demonstrated a lack of benefit of adjuvant 5-fluorouracil (FU)-based chemotherapy in stage II CRC patients with dMMR status [26]. Therefore, patients with stage II CRC with dMMR status and high-risk factors are more likely to benefit from combination chemotherapy.

However, this study still has some limitations. First, this is a retrospective study comprising a limited number of patients at a single center. A future multicenter study with a larger patient population is needed to evaluate the external utility of this nomogram. Second, due to the characteristics of retrospective studies, some useful information was missing in this study. For instance, it is not clear which kind of adjuvant chemotherapy the stage II CRC patients in the current study received and how of the types of chemotherapies were distributed among groups. Third, the 1-year AUC value of the nomogram based on the validation set was 0.673, which suggests that external cohorts are required to validate the reliability of our nomogram. Additional prospective data collection and the incorporation of other factors are encouraged to improve this model.

## Conclusion

In conclusion, we established and validated a nomogram for predicting the personalized survival probability of stage II CRC patients. This convenient nomogram had a sufficient ability to discriminate patients, in addition to excellent clinical utility, suggesting that it could be a potential simple-to-use tool for physicians to facilitate postoperative personalized prognostic evaluation and determine therapeutic strategies for stage II CRC patients.

## Supplementary information

**Additional file 1: Table S1.** Point assignments and predictive scores for each variable in the nomogram model.

**Additional file 2: Figure S1.** Subgroup analyses based on LNH status, perineural invasion status, T stage and MMR status.

## Data Availability

The dataset used during the study are available from the corresponding author on a reasonable request.

## Abbreviations

CRC: Colorectal cancer
AJCC/UICC: American Joint Commission on Cancer/International Union against Cancer
TNM: Tumor-node-metastasis
MSI: Microsatellite instability
dMMR: Mismatch repair deficiency
MSS: Microsatellite stable
pMMR: Proficient MMR
FUSCC: Fudan University Shanghai Cancer Center
DCA: Decision curve analysis
Pre-CEA: Pretreatment carcinoembryonic antigen
CRM: Circumferential resection margin
LNH: Lymph nodes harvested
C-index: The concordance index
ROC: Receiver operating characteristic
DFS: Disease-free survival
ASCO: The American Society of Clinical Oncology
EGTM: The European Group on Tumor Markers
