# Supplementary material for: Establishment and validation of a novel nomogram incorporating clinicopathological parameters into the TNM staging system to predict prognosis for stage II colorectal cancer
Source: Cancer Cell Int. 2020 Jul 6;20:285. doi: 10.1186/s12935-020-01382-w (PMC7339452; doi:10.1186/s12935-020-01382-w)
Supplement: Supplementary file 1 — Additional file 1: Table S1. Point assignments and predictive scores for each variable in the nomogram model. [file 12935_2020_1382_MOESM1_ESM.docx]

| Table S1. Point assignments and predictive scores for each variable in the nomogram model |
| --- |

| Variables | Classification | Nomogram score |
| --- | --- | --- |
| CEA | Negative | 0 |
|  | Positive | 48.70 |
| Age at diagnosis | <60 | 0 |
|  | ≥60 | 42.52 |
| Perineural invasion | Negative | 0 |
|  | Positive | 43.22 |
| CRM status | Negative | 0 |
|  | Positive | 99.48 |
| LNH | ≥12 | 0 |
|  | <12 | 48.54 |
| Differentiation | Well | 0 |
|  | Moderate | 44.97 |
|  | Poor | 89.95 |
| MMR status | dMMR | 0 |
|  | pMMR | 49.86 |
| T stage | T3 | 0 |
|  | T4a | 28.32 |
|  | T4b | 100 |
| CEA, carcinoembryonic antigen; CRM, circumferential resection margin; LNH, lymph node harvested; MMR, mismatch repair | | |
