# Supplementary figures and images for: Establishment and validation of a novel nomogram incorporating clinicopathological parameters into the TNM staging system to predict prognosis for stage II colorectal cancer
Source: Cancer Cell Int. 2020 Jul 6;20:285. doi: 10.1186/s12935-020-01382-w (PMC7339452; doi:10.1186/s12935-020-01382-w)

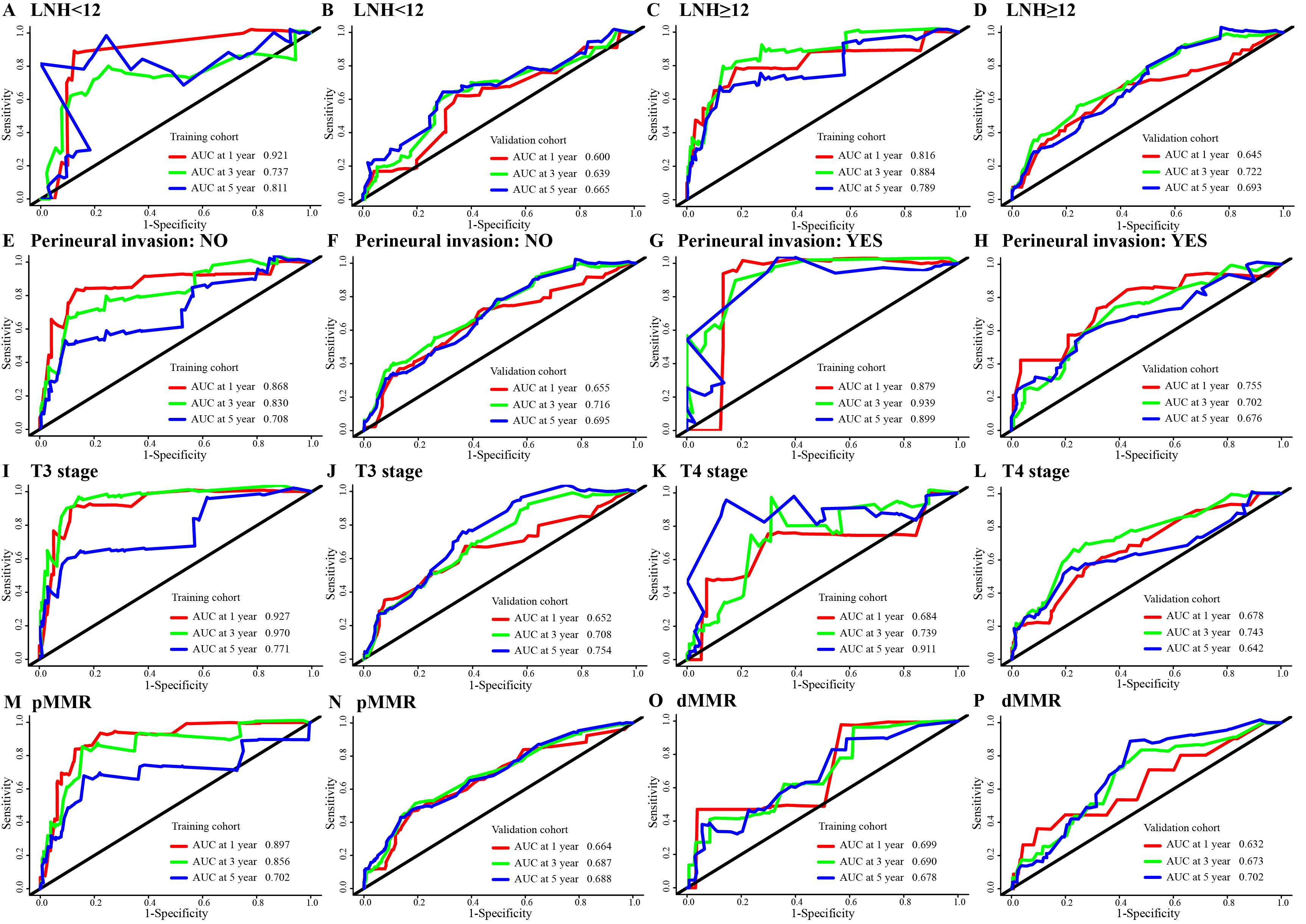

Supplement: Supplementary file 2 — Additional file 2: Figure S1. Subgroup analyses based on LNH status, perineural invasion status, T stage and MMR status. [file 12935_2020_1382_MOESM2_ESM.jpg]
